# Methyl-Donor and Cofactor Nutrient Intakes in the First 2–3 Years and Global DNA Methylation at Age 4: A Prospective Cohort Study

**DOI:** 10.3390/nu10030273

**Published:** 2018-02-27

**Authors:** Rachael M. Taylor, Roger Smith, Clare E. Collins, David Mossman, Michelle W. Wong-Brown, Eng-Cheng Chan, Tiffany-Jane Evans, John R. Attia, Tenele Smith, Trent Butler, Alexis J. Hure

**Affiliations:** 1 Priority Research Centre for Reproductive Science, University of Newcastle, Callaghan, NSW 2308, Australia; roger.smith@newcastle.edu.au (R.S.); clare.collins@newcastle.edu.au (C.E.C.); tenele.smith@newcastle.edu.au (T.S.); trent.butler@newcastle.edu.au (T.B.); 2 Faculty of Health and Medicine, School of Medicine and Public Health, University of Newcastle, Callaghan, NSW 2308, Australia; cheng.chan@newcastle.edu.au (E.-C.C.); john.attia@newcastle.edu.au (J.R.A.); alexis.hure@newcastle.edu.au (A.J.H.); 3 Hunter Medical Research Institute, 1 Kookaburra Circuit, New Lambton Heights, NSW 2305, Australia; david.mossman@uon.edu.au (D.M.); michelle.wong-brown@newcastle.edu.au (M.W.W.-B.); tiffany.evans@hmri.org.au (T.-J.E.); 4 Faculty of Health and Medicine, School of Health Sciences, University of Newcastle, Callaghan, NSW 2308, Australia; 5 Priority Research Centre in Physical Activity and Nutrition, University of Newcastle, Callaghan, NSW 2308, Australia; 6 Department of Molecular Medicine, NSW Health Pathology, John Hunter Hospital, New Lambton Heights, NSW 2305, Australia; 7 Faculty of Health, School of Biomedical Sciences and Pharmacy, University of Newcastle, Callaghan, NSW 2308, Australia; 8 Clinical Research Design IT and Statistical Support (CReDITSS) Unit, Hunter Medical Research Institute, 1 Kookaburra Circuit, New Lambton Heights, NSW 2305, Australia; 9 Priority Research Centre for Generational, Health and Ageing, University of Newcastle, Callaghan, NSW 2308, Australia

**Keywords:** child diet, global DNA methylation, nutrition, one-carbon metabolism, postnatal

## Abstract

Background: During the early postnatal period, the impact of nutrition on DNA methylation has not been well studied in humans. The aim was to quantify the relationship between one-carbon metabolism nutrient intake during the first three years of life and global DNA methylation levels at four years. Design: Childhood dietary intake was assessed using infant feeding questionnaires, food frequency questionnaires, 4-day weighed food records and 24-h food records. The dietary records were used to estimate the intake of methionine, folate, vitamins B2, B6 and B12 and choline. The accumulative nutrient intake specific rank from three months to three years of age was used for analysis. Global DNA methylation (%5-methyl cytosines (%5-mC)) was measured in buccal cells at four years of age, using an enzyme-linked immunosorbent assay (ELISA) commercial kit. Linear regression models were used to quantify the statistical relationships. Results: Data were collected from 73 children recruited from the Women and their Children’s Health (WATCH) study. No association was found between one-carbon metabolism nutrient intake and global DNA methylation levels (*P* 0.05). Global DNA methylation levels in males were significantly higher than in females (median %5-mC: 1.82 vs. 1.03, males and females respectively, (*P* 0.05)). Conclusion: No association was found between the intake of one-carbon metabolism nutrients during the early postnatal period and global DNA methylation levels at age four years. Higher global DNA methylation levels in males warrants further investigation.

## 1. Introduction

DNA methylation is an epigenetic mechanism that involves the transfer of a methyl group (−CH_3_) to a DNA cytosine-phosphate-guanine (CpG) dinucleotide. The establishment and maintenance of DNA methylation patterns is essential for mammalian development and physiological function in the adult organism [1]. Following fertilisation, most DNA methylation patterns are erased and re-established after implantation [2,3,4]. During embryogenesis and early postnatal life, DNA methylation increases, leading to cell differentiation and organogenesis [5,6,7]. De novo methylases DNMT3a and DNMT3b [8] are responsible for establishing DNA methylation patterns and DNMT1 [9] is responsible for maintaining those patterns during cellular divisions. DNA methylation modifications occurs throughout development in response to internal and external signals triggered by the environment. Mis-programming of methylation patterns during critical windows of development may alter cell and tissue functioning, thereby increasing human disease susceptibility later in life [10,11,12,13].

**Figure 1:**
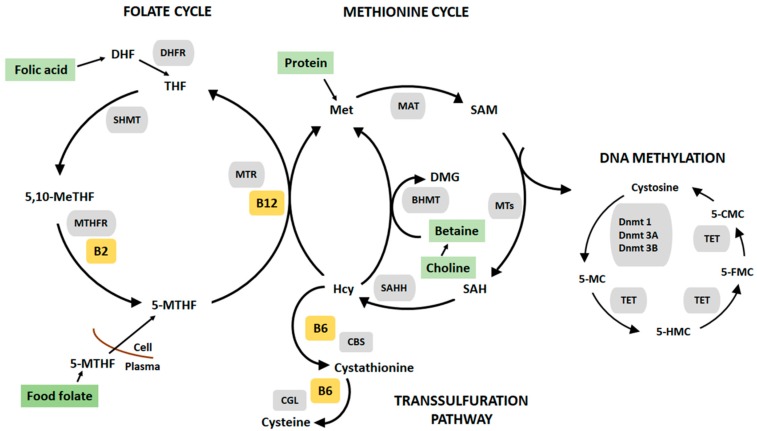
The one-carbon metabolism and DNA methylation. The Folate cycle: folic acid (supplement form) is reduced to dihydrofolate (DHF) and tetrahydrofolate (THF) by the enzyme dihydrofolate (DHFR). The carbon of serine is transferred to THF by serine hydroxyl-methyltransferase (SHMT), 5,10-methylene tetrahydrofolate (5,10-MeTHF) can be reduced to 5-methyl tetrahydrofolate (5-MTHR metabolised from food folate) by methylenetetrahydrofolate reductase (MTHFR) with vitamin B2 serving as a cofactor. The Methionine cycle: 5-MTHR serves as a methyl donor in the conversion of amino acid, homocysteine (Hcy) to methionine (Met), catalysed by methyltetrahydrofolate-homocysteine methyltransferase (MTR) and vitamin B12 serves as a cofactor. Alternatively, homocysteine can be remethylated by the enzyme betaine homocysteine methyltransferase (BHMT) and betaine, producing methionine and dimethylglycine (DMG). Betaine can be derived directly from the diet or converted from dietary choline. Methionine is metabolized to S-S-adenosylmethionine (SAM) by the enzyme methionine adenosyltranferase (MAT). SAM can also be converted to S-adenosylhomocysteine (SAH) by methyltransferase (MT). SAH is converted to homocysteine by S-adenosylhomocysteine hydrolase (AHCY). The Transulfuration pathway: Homocysteine can also be metabolized to cysteine via the actions of two vitamin B6-dependent enzymes cystathionine beta synthase (CBS) and cystathionine gamma lyase (CGL). DNA methylation: A family of enzymes called DNA methyltransferase (Dnmts) catalyses the transfer of methyl groups from SAM to DNA cytosine bases at the 5th carbon (5-mC). Active demethylation of 5-mC by ten-eleven translocation (TET) enzymes producing 5-hydromethylcytosine (5-HMC), then formylcytosine (5-FMC) and lastly 5-carboxylcytosine (5-CMC).

DNA methylation occurs via the one-carbon metabolism pathway (Figure 1), which involves the transfer of methyl groups from one site to another via a cascade of biochemical reactions. Nutrition during pregnancy and postnatal life can significantly affect DNA methylation, since methyl groups and cofactors of one-carbon metabolism are derived from nutrients supplied in the diet including, methionine (amino acid), folate (vitamin B9), choline, betaine, riboflavin (vitamin B2), pyridoxine (vitamin B6) and cobalamin (vitamin B12) [14]. Food sources rich in these nutrients are shown in Table 1.

One-carbon metabolism nutrients consumed in the diet are absorbed in the small intestine by active or passive transport mechanisms and are then released into the portal circulation. At the cellular level, folate metabolism occurs in the mitochondria, while the remaining one-carbon metabolism pathways occur in the cytosol. Single nucleotide variants or polymorphisms in specific genes can alter the efficiency of biochemical pathways in one carbon metabolism. For example, polymorphism C677T locus (cytosine (C) is replaced by thymine (T)_at base position 677) in the methylenetetrahydrofolate reductase (MTHFR) gene impairs folate and homocysteine metabolism and DNA methylation capacity, as well as altering disease risk [15]. Therefore, the nutrient requirements for one-carbon metabolism may vary according to specific genotypes.

One-carbon metabolism nutrients are necessary for the production of S-adenosyl-methionine (SAM), the universal methyl donor, required for DNA methylation [16,17]. The addition of a methyl group to a DNA dinucleotide regulates gene expression by preventing the binding of transcription factors or through the recruitment of proteins that bind to methylated DNA and alter chromatin configuration [18,19]. Therefore, a nutritional deficiency or excess may induce aberrant DNA methylation patterns (hypo- or hypermethylation) that adversely affect gene activation and disease susceptibility [20,21,22].

The effects of in utero nutrition on DNA methylation patterns of the offspring have been explored in both animal and human models. The classic ‘Agouti mouse model’ has shown that supplementing the diet of pregnant dams with one-carbon metabolism nutrients (choline, betaine, folic acid, vitamin B12, methionine and zinc) resulted in offspring with a brown coat colour and lower incidence of obesity, cancer and diabetes, which was attributed to the hypermethylation of the agouti viable yellow (Avy) locus agouti gene [23]. This animal model has shown that modifying the maternal availability of one-carbon metabolism nutrients can affect DNA methylation patterns and offspring health outcomes, which has led to further studies in humans. In the Gambia, a country in west Africa, children conceived during the nutritionally disadvantaged, rainy season, had higher DNA methylation at metastable epialleles (loci) compared to children conceived during the dry season when food was more abundant, suggesting that subtle changes in nutrient availability can alter DNA methylation patterns [24]. Further analysis confirmed that differences in nutrient intake may explain the differences in DNA methylation patterns, since maternal dietary intakes of betaine, folate and riboflavin, significantly varied between the rainy and dry seasons [25]. The impact of these DNA methylation patterns on the infant phenotype is not yet known. Overall, the findings of these animal and human studies suggest that the supply of one-carbon metabolism nutrients influences DNA methylation. Variations in DNA methylation patterns may be explained by altered gene expression of DNMTs. For example, a study in rodents demonstrated that a choline deficiency during pregnancy results in hypomethylation of the regulatory CpGs within the DNMT1 gene in the liver of embryos. This led to the up-regulation of DNMT1 and resulted in increased global and insulin growth factor DNA methylation in the liver [26].

Our understanding of the role of early postnatal nutrient intake and DNA methylation patterns is in its infancy. Following birth, infant feeding in Western countries varies considerably from exclusive breastfeeding or formula feeding to a combination of both. The Women and their Children’s Health (WATCH) study, a small but detailed prospective longitudinal birth cohort, found that breastfed infants had lower plasma B12 and folate and higher homocysteine levels compared to formula fed infants at six months of age [27]. However, the impact of these findings on DNA methylation patterns at the gene regulation level, remains unclear. Martino et al. [28] demonstrated that widespread DNA methylation changes occur across the genome in buccal cells between birth and 18 months in monozygotic and dizygotic twin pairs, in response to human development and environmental exposures. DNA samples collected using buccal swabs are often used for infants and young children because the collection method is the least invasive and the cell population is more homogenous compared to blood samples [29]. Pauwels et al. [30] recently reported higher CpG methylation levels at six months in infants whose mothers had a higher dietary choline intake (≥275 mg/day) and who were breastfed for at least three months. This field of research highlights that DNA methylation modifications can occur in the postnatal period and infant nutrition may be an important driver of DNA methylation patterns.

This study investigated the association of: (1) Cumulative nutrient intake (methionine, folate, vitamin B2, B6, B12 and choline) during infancy and early childhood (three months to three years) on global DNA methylation in buccal cells at four years of age; (2) Nutrient intake (methionine, folate, vitamin B2, B6, B12 and choline) at three years of age on global DNA methylation in buccal cells at four years of age.

## 2. Materials and Methods

### 2.1. Study Population

**Figure 2:**
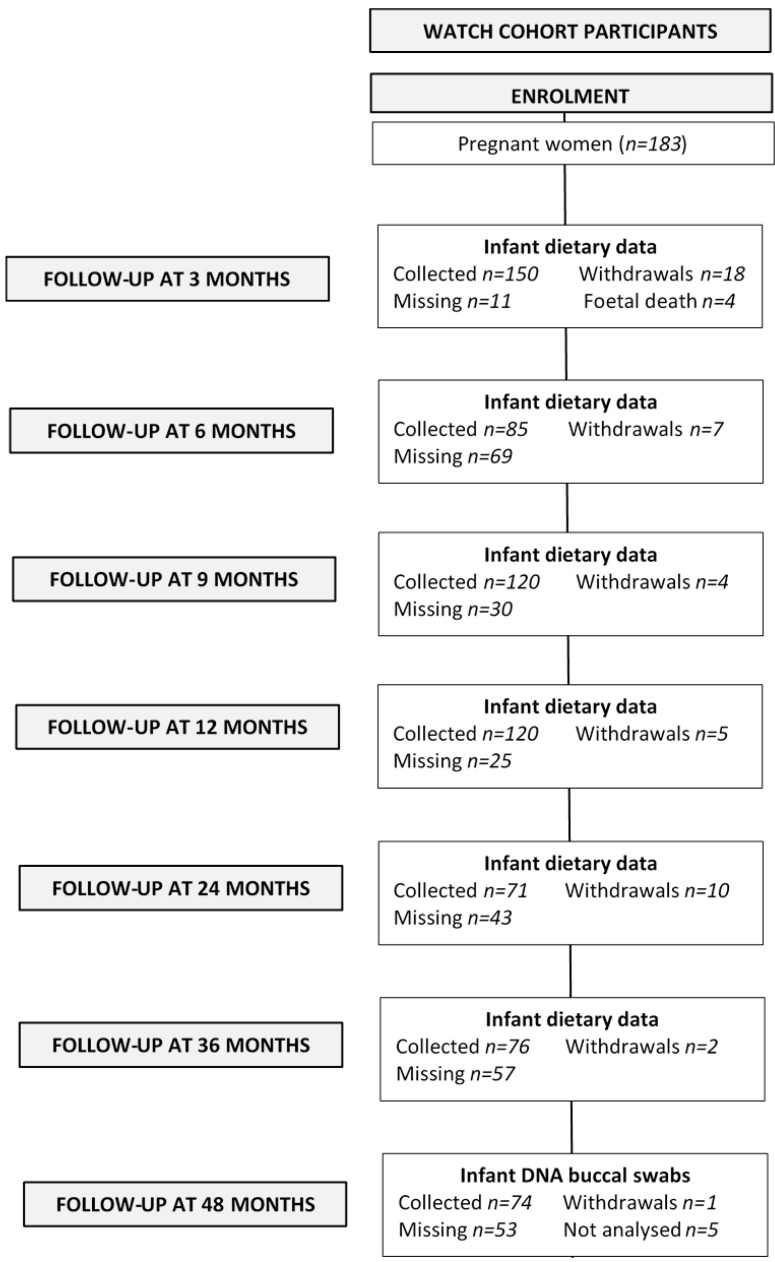
Flowchart of mother-child pairs enrolled in the WATCH cohort and included in the statistical analysis.

This study analysed data for children and mothers enrolled in a prospective, longitudinal cohort, called the WATCH study [31]. Briefly, pregnant women were recruited from the antenatal clinic at the John Hunter Hospital (JHH), New South Wales (NSW), Australia, from July 2006 to December 2008. All women who were less than 18 weeks pregnant, lived in the local or neighbouring areas and were able to attend JHH were eligible to participate. Women were recruited by midwives and through local media coverage, or by word of mouth. A consent rate of 61% was achieved for the pregnant women who were approached to participate in the study and 181 women were enrolled in the study. Pregnant women attended study visits at approximately 19, 24, 30 and 36 weeks’ gestation. The mothers and their offspring continued to attend study visits at quarterly intervals for the first 12 months after birth and then annually until four years of age. Study visit attendance and participant withdrawals are reported in Figure 2. The WATCH study received ethics approval from the Hunter New England Research Ethics Committee (06/05/24/5.06) and all participants gave written informed consent.

### 2.2. Dietary Assessment

Dietary intake was assessed using a variety of methods, which have been previously reported [31] including two different infant feeding questionnaires, 24-h dietary recalls (24hr-DR), food frequency questionnaires (FFQ) and four-day weighed food records (4d-WFR).

#### 2.2.1. Infant Feeding Questionnaires

Infant feeding data were reported by the mothers (or father of one child) and recorded by an Accredited Practising Dietitian (APD) at three-month intervals during the first postnatal year and at two years of age. The Infant Feeding Recall questionnaire was used to record breastfeeding initiation, duration (ever, at hospital discharge and currently) and exclusivity in weeks. Further questions were asked about the infant’s consumption of formula, cow’s milk and other milk substitutes and if/when semi-solid or solid foods were introduced. The questions were selected from the 2001 NSW Child Health Survey [32] and the 1995 National Nutrition Survey [33] as suggested by Hector et al. [34].

The Current Feeding Practices questionnaire recorded the infant’s breastfeeding and formula intake and whether the infant received any solids or semi-solid foods, fluids and/or dietary supplements within the previous 24 h. The Current Feeding Practices questionnaire was based on the national breastfeeding monitoring recommendations of Webb et al. [35]. The World Health Organization (WHO) breastfeeding definitions [36] were used to classify the infants as being exclusively, predominantly, complementarily, or not breastfed at each study visit.

#### 2.2.2. 24-h Dietary Recalls

An APD administered a structured 24hr-DR interview to quantify the child’s total dietary intake at ages 9 and 12 months and 2 and 3 years. The mothers recalled their child’s dietary intake of all foods, beverages and dietary supplements from the previous day or in person at a subsequent hospital visit.

#### 2.2.3. Four-day Weighed Food Records

The 4d-WFRs were completed by the mothers for their offspring at ages six months and at 2–3 years. The child’s food and beverage consumption was recorded on three consecutive or non-consecutive days and one weekend day within two weeks of their study visit. The mothers were provided with SOEHNLE Venezia electronic scales (Soehnle-Waagen GMbH Co., Murrhardt, Germany) to measure the food and beverages consumed on the specified days. Food diaries were provided and written instructions were given to the mothers for the recording of their child’s dietary intake. The mothers already had experience in completing the 4d-WFR from their pregnancy study visits. The completed food diaries were returned in a reply-paid envelope or in person to the researchers.

#### 2.2.4. Food Frequency Questionnaire

The Australian Child and Adolescent Eating Survey (ACAES) (36) FFQ was used to measure dietary intake of children from two years of age. The ACAES is a 135-item semi-quantitative FFQ, with 15 supplementary questions including, age, nutrient supplement use, food behaviours and sedentary behaviours, developed to measure dietary intake over the previous six months of children aged 2 to 17 years [37]. The survey has demonstrated acceptable accuracy for ranking nutrient intake in Australian youths aged 9 to 16 years [37]. The WATCH mothers reported their child’s consumption of a food item over the previous six months, using frequency categories ranging from never to four or more times per day. Portion sizes for individual food items were recorded using “natural” serving size (e.g., a slice of bread) or were determined from the 1995 National Nutrition Survey [33]. Further questions were asked about the total number of daily servings of fruit, vegetables, bread, dairy products, eggs, fat spreads, sweetened beverages and snack foods, as well as the type of bread, dairy products and fat spreads used. The survey contained 12 questions relating to eating behaviours, including fast-food consumption and eating while watching television.

### 2.3. Dietary Analysis

#### 2.3.1. Quantifying Energy and Nutrient Intake from Breastmilk

The breastmilk intake of the WATCH infants was estimated using their estimated energy requirements (EER) and actual energy intake (EI) recorded in FoodWorks. The EER was calculated in FoodWorks using The Food and Nutrition Board: Institute of Medicine [38] age specific equations, which include infant requirements for energy deposit. The EI was the total daily energy consumed by the child, as reported in the infant feeding questionnaires, 24hr-DR and 4d-WFR. For exclusively breastfed infants their energy intake was equal to their EER. For infants who consumed solid food and/or formula, as well as breastmilk, their energy intake from breastmilk was calculated using the difference between the infant’s EER and EI. The volume (mL) of breastmilk consumed was calculated by dividing the total energy from breastmilk by three (i.e., breastmilk 1 mL = 3 KJ). This method has demonstrated acceptable accuracy for estimating infants’ energy intake from breastmilk [39].

#### 2.3.2. Dietary analysis of Protein, Folate, Vitamin B2, B6 and B12 Intake

An APD entered the dietary records into the nutrient-analysis software, Foodworks 7, 2012 (Xyris Software (Australia) Pty Ltd., Brisbane, QLD, Australia), which includes the current food composition data used in Australia (AUSNUT 2013 [40], AUSNUT 2007 [41], NUTTAB 2010 [42]). The Australian Nutrition Tables (NUTTAB 2010) nutrient composition data for each food item is primarily derived from Australian laboratory food analysis [42]. The Australian Nutrition Tables (AUSNUT 2013) provides more comprehensive data for the nutrients vitamin B6 and B12, for foods and beverages consumed during the 2011–2013 Australian Health Survey [40]. The AUSNUT 2007 estimates the nutrient composition of foods, beverages and dietary supplements consumed by children aged 2–16 years in the 2007 National Children’s Nutrition and Physical Activity Survey [43]. The database contains 37 nutrient values (excluding vitamins B12 and B6) for 4225 foods, beverages and dietary supplements [41].

The Australian food databases were used to quantify the WATCH children’s mean energy and nutrient intakes, including, protein, folate, vitamin B2, B6 and B12. Foods that could not be found in the Australian food databases were supplemented with data from food recipes provided by study participants. For example, a recipe for strawberry coconut muffins was provided by one study participant and the recipe and portioning was analysed using the Australian food databases to determine the nutrient composition of the single serving consumed by the child.

In Australia, mandatory food (bread) fortification of folic acid and iodine was introduced by Food Standards Australia and New Zealand (FSANZ) in 2009 [44,45]. The WATCH dietary data were collected from 2006 to 2011 which coincided with the transition from voluntary to mandatory food fortification. Therefore, the quantified mean daily intake of folate from the WATCH dietary records included both naturally occurring and synthetic nutrient sources during that time period.

#### 2.3.3. Dietary Analysis of Methionine and Choline

National food composition data for methionine and choline are limited. Consequently, the habitual intake of these nutrients at a population level is not well documented and the following protocol was used for estimating these nutrients.

#### 2.3.4. Methionine

Quantifying the daily intake of methionine from the dietary records was achieved using the NUTTAB 2010 amino acids database Excel file. The Australian database only quantifies the methionine content of 98 food items, therefore supplementary data were obtained from the United States Department of Agriculture (USDA) National Nutrient Database for Standard Reference, release 28 (2016) [46] and the Canadian Nutrient File (CNF), 2015 edition [47]. The USDA provides methionine data values for 5053 food items [46] and the CNF provides methionine data values for up to 5898 food items [47]. If a food item could not be found in any of the databases, a comparable food was used based on the ingredient list provided by the food manufacturer and the nutrient composition provided in the database. For example, a Kellogg’s K-Time Baked Twist (cereal bar with strawberry and blueberry filling) could not been found in the food databases, therefore nutrient data from a fruit filled granola bar (found in the CNF food database) was used as an alternative since the protein content differed by only one gram between the two food items of the same quantity.

Internationally, most infant formula manufacturers do not report the methionine content of their product and analytical data are not widely available [48,49]. Therefore, the amount of methionine consumed from infant formula in WATCH was estimated using the quantified values reported by Agostoni et al. [48]. The methionine values reported for the cow’s milk-based formula, Aptamil and the soy-based formula, Isomil were used [48]. The number of food and beverage items from each food database used to quantify methionine intake for all the WATCH dietary records is provided in Supplementary Figures S1–S3.

#### 2.3.5. Choline

To date no Australian food databases have quantified the choline content of Australian foods, therefore the USDA database for the choline content of common foods, release 2, 2008 [50] and the CNF database were used. The USDA database provides the choline content of 4680 food items [50] and the CNF database provides the choline of 2774 food items. If a food item could not be found in either database, a comparable food was used based on the ingredient list provided by the food manufacturer and the nutrient composition provided in the database. For example, a savoury wheat-based cracker/crispbread (SAO) could not be found in the food databases, nutrient data from regular wheat crackers (found in the USDA food database) were used because the macronutrients only varied by 1–4 g between the two food items of the same quantity.

The choline content of infant formulas was quantified using the values reported by the formula manufacturer. The mean choline value of 11 Australian formula brands was used for formula manufacturers that did not report the choline content of the product. The number of food and beverage items from each food database used to quantify choline intake for all the WATCH dietary records is provided in Supplementary Figures S1–S3.

#### 2.3.6. Quantifying Nutrient Intake from FFQs

Nutrient intakes from the FFQ data were estimated by multiplying the quantity (grams) of a food item consumed per day by the nutrient content of that food item (per grams) reported by Australian food databases for methionine, folate, vitamin B2, B6 and B12 and the USDA database for methionine and choline. The combined total of all the food items was used to estimate the child’s intake of methyl-donor and cofactor nutrients from the FFQs.

#### 2.3.7. Maternal Supplementation

Pauwels et al. [30] recently reported that maternal supplemental intake during the preconception period, was associated with lower (−0.706% decrease) buccal cell DNA methylation in the gene, insulin-like growth factor 2 (IGF2) in six-month-old infants. In addition, previous findings from the WATCH study have demonstrated that the mothers’ mean plasma folate concentrations during pregnancy were correlated with the infants’ homocysteine concentrations at six months of age, after adjustment for the infants’ own folate concentrations. Furthermore, Joubert et al. [2] reported that higher maternal plasma folate concentrations during pregnancy were associated with decreased methylation of 416 CpGs and increased methylation of 27 CpGs in the offspring at birth. In light of these findings, the current study analysed the association between maternal supplement intake and child buccal cell DNA methylation at a global level. Information about the WATCH mother’s nutrient supplement use (type/brand, frequency, dosage and duration) during the preconception period and pregnancy was collected during their 19-week study visit via interview.

### 2.4. DNA Collection

DNA samples were collected from the WATCH children at four years of age using buccal cheek swabs. In preparation for the cheek swabs, the children refrained from eating and drinking 45 min prior to their study visit, while other study data were collected. Using the Isohelix buccal DNA isolation kits and protocol (Cat. no. SK-1S, Isohelix, Harrietsham, Kent, UK), a research assistant firmly rubbed a sterile swab head against the child’s inside cheeks for approximately 20 s on both sides, the swab was placed in a sterile 5 mL tube and stored at −80 °C Buccal cheek cell samples were chosen for DNA methylation analysis because they are the least invasive sample to collect in a cohort of young children and the cell population is more homogenous compared to blood samples [52]. Compared with blood, buccal samples have also shown greater correlation in the hypomethylated tissue-specific differentially methylated regions (tDMRs) with hypomethylated regions in other tissues (brain, full-term placenta, liver, kidneys, pancreas, skeletal muscle and sperm) [52].

### 2.5. DNA Extraction

The Qiagen Gentra Puregene Buccal Cell Kit (cat no. 158845, Qiagen, Valencia, CA, USA) was used to extract DNA from the buccal cells. The following steps were performed according the manufacturers’ instructions. The swab heads were removed from the handle and added to 3 mL tubes containing 300 µL of cell lysis solution and 1.5 µL proteinase K. After incubating at 55 °C overnight, the swab heads were discarded and 1.5 µL RNase A solution was added to the tubes. The DNA samples were incubated at 37 °C for one hour prior to adding 100 µL protein precipitation solution to the tubes. Precipitated proteins and insoluble cellular debris were pelleted by centrifuging the 2 mL tubes at 13,000× *g* for three minutes and incubating them on ice for five minutes. This step was repeated to form a tight protein pellet. The supernatant was collected into a sterile 1.5 mL tubes and 300 µL of isopropanol and 0.5 µL of glycogen solution were added to precipitate the DNA. The tubes were gently inverted and centrifuged at 13,000× *g* for five minutes. The supernatant was discarded and the DNA pellets were re-suspended in 300 µL of ethanol solution (70%) and centrifuged at 13,000× *g* for one minute. The supernatant was discarded and the DNA pellets were left to dry at room temperature for five minutes. The DNA pellets were resuspended in 20 µL of DNA hydration solution and centrifuged for three minutes at 13,000× *g*. The DNA samples were incubated at room temperature overnight. The DNA concentration of each sample was quantified using the NanoDrop1000 (Thermo Fisher Scientific, San Jose, CA, USA).

### 2.6. Quantification of Global DNA Methylation

Previous studies, have reported an association between global DNA methylation patterns and human disease states [53,54,55]. Therefore, the current study analysed the impact of postnatal nutrition on global DNA methylation, to determine if this is an important determinant of future health and disease susceptibility. Global DNA methylation of the child buccal cheek swabs was analysed using an (indirect) ELISA-based commercial Kit (MethylFlash Methylated DNA 5-mC Quantification Kit (Colorimetric), Epigentek Group Inc., New York, NY, USA, cat. no. P-1034-96). In the assay, 0.4–5 µL of sample DNA (25–100 ng input DNA) was bound to strip wells with a high DNA affinity. Methylated DNA was detected using capture and detection antibodies to 5-methylcytosine (5-mC) and then quantified colorimetrically by reading the absorbance at 450nm, using a Spectrostar Nano plate reader (BMG Labtech, Ortenberg, Baden-Wurttemberg, Germany). In this ELISA, the amount of methylated DNA is proportional to the optical density (OD). In human somatic cells, 70–80% of CpG dinucleotides are methylated, which constitute to less than 1% of the genome [56]. Therefore, the percentage of detected 5-mC is expected to be low, due to the low prevalence of CpGs in the human genome. All DNA samples were analysed in triplicates; however, if the amount of DNA was limited, the samples were analysed in duplicate and mean values were used for the statistical analysis. A standard curve was generated according to the manufacturer’s instructions and was used to quantify the percentage of methylated DNA in the total DNA sample.

### 2.7. Participant Characteristics

Sociodemographic, maternal and medical information was collected from the WATCH mothers during their first study visit. Sociodemographic information included residential postcode, which was converted to a ranking relative to socio-economic advantage and disadvantage within NSW using data from the Australian Bureau of Statistics (ABS) Socio-economic Indexes for Areas (SEIFA) [57]. The mother’s level of education attainment (no formal qualifications, year 10 school or equivalent, year 12 school or equivalent, trade/apprenticeship, certificate/diploma, university degree or higher university degree) personal and household level of income (no income, AU$1–119/wk., AU$120–299/wk., AU$300–499/wk., AU$500–699/wk., AU$700–999/wk., AU$1000–1499/wk., AU$1500/wk. or more) and marital status (never married, married, separated, divorced or widowed) were also collected.

Maternal age and body mass index (BMI) during pregnancy (normal weight (18.5–24.99 kg/m^2^), overweight (25–29.99 kg/m^2^), or obese (≥30 kg/m^2^)) were also collected. The NSW Health ObstetriX electronic database [58] was accessed to retrieve the history of the mother’s previous pregnancies and medical history and neonatal anthropometry data, including birth weight, length and head circumference.

### 2.8. Statistical Analysis

To analyse the WATCH dietary data, the intake of methyl-donor and cofactor nutrients was ranked into quintiles for each of the six time points (from 3 to 36 months postnatally). A quintile rank of five was the highest level of nutrient intake. When nutrient values were missing at specific time points, imputed values were used based on the participant’s mean nutrient rank. If nutrient data were missing for more than three time points the study participant was excluded from analysis. At all time points, nutrient data were obtained from the most reliable dietary collection method. The reliability of the dietary collection methods was prioritised from highest to lowest: 4d-WFR, FFQ, 24hr-DR and infant feeding questionnaire. For each nutrient, a summary measure of the cumulative nutrient intake over the study period was calculated by summing the nutrient ranks across all time points for each study participant.

Linear regression was used to model the association of cumulative nutrient intake with global DNA methylation and the association of nutrient intake at three years of age with global DNA methylation. The natural logarithm was used for the outcome variable, DNA methylation, in order to minimise the influence of outliers. The effect of nutrient intake is reported as the expected change in the natural logarithm transformation of global DNA methylation percentage for each unit increase in nutrient quintile rank. An indicator variable for pregnancy supplementation was included in the regression to analyse if this variable was associated with child DNA methylation. Each linear regression was adjusted for child gender. All tests assumed a 5% significance level. All statistical analyses were performed using Statistical Analysis System (SAS) software (version 9.4, SAS Institute, Cary, NC, USA).

## 3. Results

Seventy-three children from the WATCH cohort, provided dietary information and buccal DNA samples (41 female, 32 male). Global DNA methylation could not be analysed in four samples and six samples could only be analysed in duplicates rather than triplicates due to limited (0.5ng per 1 mL) DNA available. Children with dietary information at three or more out of the six time-points were included in the analysis; seven children that did not meet this criterion were excluded from the analysis. Dietary intake was available for approximately 23% (*n =* 17) of the children at six time-points, 38% (*n =* 28) at five time-points, 22% (*n =* 16) at four time-points and 7% (*n =* 5) at three time-points. The main reasons for missing dietary data were that the mother and child pair missed an appointment or were not available to attend their scheduled visit. The scheduled visits for collecting dietary information was age corrected for children born preterm (37 completed weeks gestation, *n =* 6). The age range of the children at each time-point were 2–4 months for the 3-month visit, 5–7 months for the 6-month visit, 8–10 months for the 9-month visit, 11–15 months for the 12-month visit, 20–30 months for the 24-month visit and 31–41 months for the 36-month visit. A summary of the absolute nutrient intake values at each quintile and time-point is provided in Supplementary Table S1.

The characteristics of the WATCH mother-child pairs in the study subset are shown in Table 2. In summary, the mothers in this cohort tendered to be highly educated, earning a high income and married. The maternal age of the mothers ranged from 18–41 years, most women did not smoke and had 1–2 live term births including the children born in the WATCH study. The birthweights of the children ranged from 1960–5080 g.

**Figure 3:**
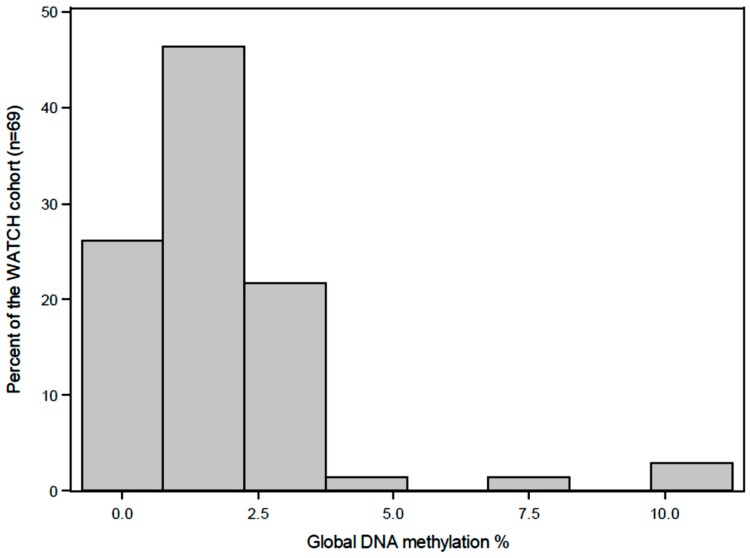
Frequency histograms for the range of global DNA methylation percentage versus percentage of the sample population for the WATCH children.

**Figure 4:**
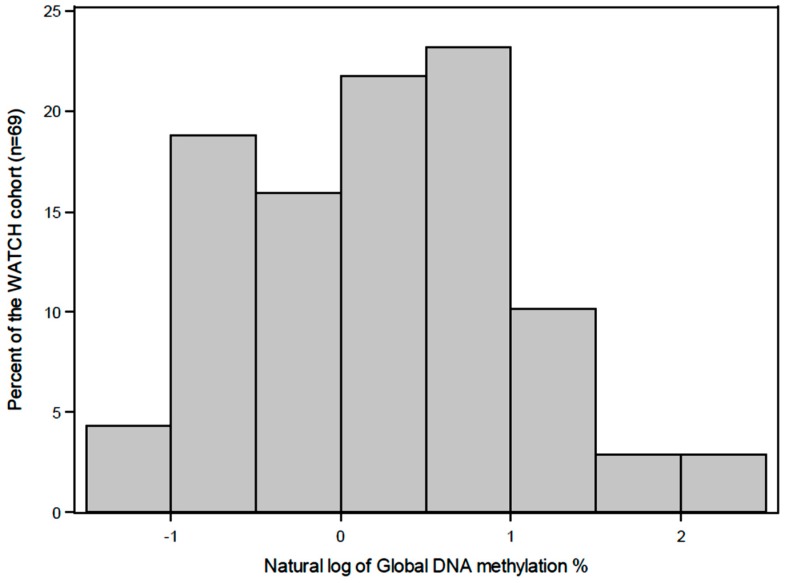
Frequency histogram for the natural logarithm transformation of global DNA methylation percentage versus percentage of the population for the WATCH children.

A preconception folic acid supplement was consumed by 42% of the mothers and 20% used a multiple micronutrient supplement that included vitamins B2, B6 and B12. During pregnancy, 41% of the mothers used a folic acid supplement and 17% used a multiple micronutrient supplement, which included vitamins B2, B6 and B12. The distribution of the global DNA methylation levels (%5-mC) in buccal cells at four years of age were skewed to the left (Figure 3) with 93% of the samples between 0–3% methylated, the median was 1.32% and the values ranged from 0.31–10.75%. Global DNA methylation levels (%5-mC) were significantly (*P* = 0.01) higher in males compares to the females (median interquartile range()) 1.82 (1.23) vs. 1.03 (1.6)). The characteristics of the children by global DNA methylation levels are summarized in Table 3, however no significant covariates could be identified. The global DNA methylation data percentage was log-transformed to satisfy normality assumptions (Figure 4).

Overall, there were no significant associations between the cumulative nutrient intake rank of vitamins B2, B6 and B12, choline, folate and methionine during the first three years of life with DNA methylation at four years in buccal cells, before or after adjustment, child gender and pregnancy supplement use (Table 4). The log transformed DNA methylation level decreased for each additional unit (maximum of 30 units) of cumulative rank of methionine, vitamin B6, choline and folate (Table 4), while the log transformed DNA methylation levels increased for each additional unit of cumulative rank of vitamins B2 and B12 (Table 4). The R-squared values for the linear regression models demonstrated that only 7 to 10% of the variation in the outcome was accounted for.

The three-year nutrient intake rank of vitamins B2, B6 and B12, choline, folate and methionine (non-cumulative) was also not statistically significantly associated with global DNA methylation at four years in buccal cells, before or after adjustment, child gender and pregnancy supplement use (Table 5). The log transformed DNA methylation level decreased for each additional unit (maximum of 30 units) of rank of methionine, vitamin B2, B6 and folate (Table 5), while the log transformed DNA methylation levels increased for each additional unit of rank of vitamins B12 and choline (Table 5).

## 4. Discussion

During postnatal life, a constant supply of dietary nutrients is necessary for one-carbon metabolism and to maintain DNA methylation patterns during multiple rounds of cellular proliferation and differentiation, that are required for organ development [59,60]. In human studies, the impact of postnatal nutrition on DNA methylation has not been well explored. Hence, the current prospective longitudinal study is important as it is one of few studies that has collected detailed dietary information during the postnatal period, especially during the first year of life and examined DNA methylation data. In addition, the intakes of nutrients involved in one-carbon metabolism were able to be quantified in this cohort. Despite these strengths, the current study did not find any statistically significant associations between the dietary intakes of methyl donor and cofactor nutrients during the first three years of life (cumulative) or at three years of age (non-cumulative) and global DNA methylation in buccal cells at four years of age. Adult disease susceptibility is likely to be a consequence of DNA methylation patterns established from the accumulation of multiple environmental exposures, including nutrition, rather than a brief exposure to a single factor [61,62,63]. However, environmental exposures during critical windows of development, may result in widespread, persistent DNA methylation changes, rather than insignificant transient changes [64,65,66].

The current study only measured DNA methylation during childhood, therefore it is not possible to distinguish how the epigenome reflects DNA methylation patterns established in utero versus the postnatal period. A longitudinal study reported that DNA methylation levels in 6641 CpG sites significantly changed from birth to five years of age [67]. Specifically, 36.79% of these sites were hypermethylated and associated with genes related to developmental functions (e.g., cell adhesion) while 63.21% were hypomethylated and associated with genes related to immune function (e.g., antigen binding) [67]. However, the specific impact of postnatal nutrition on DNA methylation levels cannot be determined from this study. Therefore, future studies that examine DNA methylation at birth and during subsequent early childhood will be highly informative for understanding the impact of prenatal and postnatal nutrition on the epigenome.

### 4.1. Impact of Postnatal Nutrition on DNA Methylation

Global DNA methylation analysis in healthy children has not been well studied. The Northern Manhattan Mothers and Newborns Study of the Columbia Centre for Children’s Environmental Health (CCCEH) cohort [68], used an ELISA-based commercial kit to analyse global DNA methylation levels in the peripheral blood of 165 children at three years of age and reported a median global DNA methylation level of 1.94%, which is 0.62% higher than the WATCH cohort. The differences in DNA methylation levels between the two cohorts are small but may be attributed to differences in the cell type. Supporting this assertion, Jiang et al. [69] recently found that overall CpG methylation was higher in peripheral mononuclear cells compared to buccal cells using the Illumina GoldenGate Assay.

Montrose et al. [70] reported that folate and choline dietary intake was associated with higher buccal long interspersed nuclear elements-1 (LINE-1) DNA methylation in asthmatic children aged 8 to 17 years, however these study findings may not be applicable to healthy children. In a large (*n* = 568) study conducted in a middle-income country, the association between micronutrient status biomarkers and LINE-1 DNA methylation in white blood cells was analysed [71]. This study did not find an association between erythrocyte folate and plasma vitamin B12 and LINE-1 methylation in children aged 5–12 years [71]. Therefore, due to the limited evidence and small samples, the association between postnatal nutrition and child DNA methylation remains to be determined and requires further characterisation. In addition, evidence from studies conducted in adults, suggest that gene variants, such as C677T MTHFR polymorphism, alter folate metabolism and diminish global DNA methylation levels [15,72]. The association between polymorphisms and global DNA methylation could be analysed in the WATCH cohort and would require further genotyping.

### 4.2. Gender Differences DNA Methylation

Global DNA methylation levels were higher in males compared to females, which is consistent with other studies [71,73,74]. Gender differences in DNA methylation patterns begin to emerge during implantation and embryo development. In females, one of the two X chromosomes is hypermethylated in the promoter regions of CpG islands, contributing to transcriptional silencing to equalise the dosage of genes between males and females. The influences of sexual differentiation during early postnatal development on DNA methylation patterns, is largely unknown. However, large hormonal changes are known to occur during postnatal life that impacts on developmental programming. In males, the testicular hormone, testosterone, peaks at 1–3 months of age and declines to baseline by about six months of age [75]. Evidence from an animal model demonstrated that an increase in the expression of DNMT3a in amygdala of the brain also coincided with the postnatal testosterone release [76]. Therefore, gender differences in DNA methylation may also be attributed to the role of sex hormones in regulating the expression of DNA methyltransferase. In addition, Ghahramani et al. [77] demonstrated the effects of testosterone in males and females treated with testosterone at birth compared to females without treatment on DNA methylation patterns was more pronounced in adulthood compared to the prenatal period. Therefore, this study suggests that the full manifestations of testosterone on the epigenome may not be evident until later in life.

### 4.3. Global and Locus Specific DNA Methylation

DNA methylation changes at a global specific level are likely to be small but still may equate to statistical significant changes in methylation at a locus specific level. Therefore, significant DNA methylation changes at a locus-specific level in the WATCH cohort cannot be excluded. For example, a recent trial analysed the impact of maternal long-chain polyunsaturated fatty acids supplementation on global and locus specific DNA methylation in the offspring at birth and five years [78]. This study did not find any differences in global DNA methylation between the intervention and control group, however group differences (5%) were identified in 21 tDMRs at birth, approximately 50% of these differences persisted until five years [78]. These findings highlight that significant differences in DNA methylation at a locus-specific level may not produce statistically significant differences at a global level. Hejimans et al. [64] demonstrated a 5.2% decrease in IGF2 in 60 years later in adults (*n =* 60) exposed to the Dutch winter famine during preconception. In addition, Pauwels et al. [30] observed that buccal DNA methylation of IGF2 in infants at six months of age was associated with their mother’s folate, folic acid and betaine intake during preconception. IGF2 is important for regulating cell growth and differentiation [79]. Analysing both global and locus-specific DNA methylation is informative for understanding what methylated regions, as well as how many methylated dinucleotides, influence gene expression and disease susceptibility risk. Therefore, DNA methylation at a locus-specific level should be analysed in the WATCH cohort.

### 4.4. Study Limitations

This cohort study was limited by its small sample size and the homogeneity of the population (i.e., generally well-nourished) and hence may explain why only able to account for 7 to 10% of the total variation in child global DNA methylation levels was accounted for by the linear modelling of nutrient intake. Due to limited statistical power, the study data may be pooled with other larger cohorts for meta-analysis to determine the role of dietary nutrients in DNA methylation. This study design could also be used as a template for the development of future studies that analyse early postnatal nutrient intake and DNA methylation.

Australian food composition data for the nutrients choline and methionine were limited, therefore, supplementary data were obtained from the US and Canada. Using food composition databases from other countries is likely to reduce the accuracy when estimating nutrient intakes and hence is indicative only. However, this study was designed to rank the children’s nutrient intake rather than estimate absolute nutrient intake, therefore it is likely that the food composition data used has ranked the children’s nutrient intake into the appropriate quintiles. Furthermore, the collection of dietary data from the WATCH cohort occurred during the transition from voluntary to mandatory folic acid fortification of the Australian food supply. Therefore, the dietary folate intake in this cohort may not be applicable to other populations and nor could we separate out the impact of fortified food products.

With unlimited resources, the whole-genome sequencing-bisulphite treatment is considered the gold standard approach, demonstrating high specificity, sensitivity and reliability [80,81,82,83]. Comprehensive data for locus-specific methylation can be obtained using microarray analysis based on sodium bisulphite modification. Therefore, the biological plausibility of our study findings should be confirmed using these techniques and compared with the results of similar studies.

## 5. Conclusions

In conclusion, this longitudinal prospective cohort did not find a statistically significant association between the dietary intake of one-carbon metabolism nutrients during the first three years of life and global DNA methylation at four years of age. Global DNA methylation levels were found to be higher in males compared to females. DNA methylation changes at a global level are likely to be small and not statistically significant, therefore locus specific DNA methylation warrants further investigation in this cohort and others. This small but detailed study was limited by its sample size and therefore only powered to detect larger changes in DNA methylation levels, however, this study can serve as a template for others and the study data may be pooled with larger cohorts for meta-analysis, to determine if there are associations in dietary methyl donors and cofactors on DNA methylation in healthy children.

## Figures and Tables

**Table 1:** Food sources rich in methyl donor nutrients and cofactors.

Nutrient	Food Sources
Choline	Cauliflower, eggs, flax seeds, lentils, liver, peanuts, soybeans and wheat germ.
Folate and folic acid	Asparagus, cheese, eggs, fortified breads and cereals, legumes, liver, peanuts, oranges and spinach.
Methionine	Dairy products, eggs, fish, meat, poultry and rice.
Vitamin B2 (Riboflavin)	Cheese, eggs, meat and milk.
Vitamin B6 (Pyridoxine)	Bananas, fish, grains, legumes, liver, meat, potatoes and poultry.
Vitamin B12 (Cobalamin)	Eggs, fish, meat, poultry, dairy products

**Table 2:** Characteristics of the WATCH mother-child pairs included in the analysis (*n =* 73).

Characteristics	Median (IQR)	Range
**Mother**		
**Maternal age (y)**	30(8)	18–41
**Education**	*n*	%
No formal qualification	1	1.4
Year 10 or equivalent	13	18
Year 12 or equivalent	13	18
Trade/apprenticeship	3	4.2
Certificate/diploma	12	17
University degree	23	32
Higher university degree	6	8.5
Missing	2	
**Income**	***n***	**%**
No income	7	9.7
$1–299	26	36
300–699	25	35
700–999	11	15
Unsure	3	4.2
Missing	1	
**Marital status**	***n***	**%**
Never married	23	32
Married	46	64
Divorced	2	2.8
Widowed	1	1.4
Missing	1	
**Maternal smoking**	***n***	**%**
Yes	7	10
No	62	90
Missing	4	
**Live births (37 weeks’ gestation)**	***n***	**%**
None	1	1.6
1–2	46	75
3–4	14	23.1
5	1	1.6
Missing	11	
**Live preterm births (≤36 weeks’ gestation)**	***n***	**%**
None	39	53.4
1	6	8.2
2	1	1.4
Missing	27	
**Child**		
**Gender**	*n*	%
Male	32	44
Female	41	56
	**Median (IQR)**	**Range**
Birth weight (g)	3560 (690)	1960–5080
Birth length (cm)	51 (4)	48–57.5
Head circumference (cm)	35 (2)	31–39.5

IQR, interquartile range.

**Table 3:** Characteristics of the WATCH children by global DNA methylation (*n* = 69).

Characteristic	Global DNA Methylation Quintiles (Minimum and Maximum)				
	1	2	3	4	5
	(0.313–0.597)	(0.598–1.036)	(1.035–1.552)	(1.554–2.611)	(2.614–10.752)
**Mother**					
Maternal age (years, median)	29	31.5	31	27	31
Maternal smoking (%)	14.3	0	15.4	0	14.3
**Children**					
Males (%)	36	21	54	43	57
Females (%)	64	77	46	57	42
Birthweight (grams, median)	3442	3560	3310	3680	3260

**Table 4:** Association of child cumulative (6-timepoints) nutrient intake rank with global DNA methylation.

Model ^1^	*N*	Nutrient ^2^	Outcome ^3^	Association (95% CI)	*P*-Value	*R*-Square
1	57	Methionine	DNA methylation %	−0.001 (−0.05 to 0.05)	0.95	0.08
2	57	Vitamin B2 (Riboflavin)	DNA methylation %	0.002 (−0.04 to 0.05)	0.94	0.08
3	57	Vitamin B6 (Pyridoxine)	DNA methylation %	−0.007 (−0.05 to 0.04)	0.74	0.08
4	57	Vitamin B12 (Cobalamin)	DNA methylation %	0.024 (−0.02 to 0.07)	0.28	0.10
5	57	Choline	DNA methylation %	−0.000 (−0.05 to 0.05)	0.99	0.07
6	57	Folate	DNA methylation %	−0.016 (−0.06 to 0.03)	0.44	0.09

^1^ All models were adjusted for child gender, models 2, 3 and 4 were for vitamin B2, B6 and B12 supplement use during pregnancy and model 6 were adjusted for folic acid supplement use during pregnancy; ^2^ The accumulative nutrient rank combines the points from each quintile (6–30 points) from the six time-points; ^3^ The natural logarithm transformation of global DNA methylation was used for the linear regression models to meet normality assumptions.

**Table 5:** Association of child nutrient intake rank at three years (1-timepoint) with global DNA methylation.

Model ^1^	*N*	Nutrient	Outcome ^2^	Association (95% CI)	*P*-Value	*R*-Square
1	57	Methionine	DNA methylation %	−0.017 (−0.18 to 0.14)	0.83	0.08
2	57	Vitamin B2 (Riboflavin)	DNA methylation %	−0.037 (−0.2 to 0.13)	0.66	0.08
3	57	Vitamin B6 (Pyridoxine)	DNA methylation %	−0.086 (−0.24 to 0.07)	0.26	0.10
4	57	Vitamin B12 (Cobalamin)	DNA methylation %	0.100 (−0.06 to 0.26)	0.23	0.10
5	57	Choline	DNA methylation %	0.037 (−0.12 to 0.19)	0.64	0.08
6	57	Folate	DNA methylation %	−0.062 (−0.21 to 0.09)	0.41	0.09

^1^ All models were adjusted for child gender, models 2, 3 and 4 were for vitamin B2, B6 and B12 supplement use during pregnancy and model 6 were adjusted for folic acid supplement use during pregnancy; ^2^ The natural logarithm transformation of global DNA methylation was used for the linear regression models to meet normality assumptions.

## Supplementary Materials

The following are available online at http://www.mdpi.com/2072-6643/10/3/273/s1, Figure S1: Food composition database used for methionine and choline quantification: 4-day weighed food records, Figure S2: Food composition database used for methionine and choline quantification: food frequency questionnaires, Figure S3: Food composition database used for methionine and choline quantification: 24-h food recall, Table S1: Quintiles of nutrient intake during early childhood in the WATCH cohort.

## Abbreviations

ABS	Australian Bureau of Statistics
ACAES	Australian Child and Adolescent Eating Survey
APD	accredited practicing dietitian
AUSNUT	Australian Nutrition Tables
Avy	agouti viable yellow
BMI	body mass index
CCCEH	Columbia Centre for Children’s Environmental Health
CNF	Canadian Nutrient File
CpG	cytosine phosphate guanine
24hr-DR	24 hour dietary recalls
DNMT	de novo methylase
EER	estimated energy intake
EI	energy intake
ELISA	enzyme-linked immunosorbent assay
FFQ	food frequency questionnaire
FSANZ	Food standards Australia and New Zealand
IGF2	insulin-like growth factor 2
JHH	John Hunter Hospital
LINE-1	long interspersed nuclear elements-1
5-mC	5-methylcytosine
MTHFR	methylenetetrahydrofolate reductase
NSW	New South Wales
NUTTAB	Nutrition tables
OD	optical density
SAM	S-adenosyl-methionine
SAS	Statistical Analysis System
SEIFA	socio-economic Indexes for Areas
tDMRs	tissue-specific differentially methylated regions
USDA	United States Department of Agriculture
WATCH	Women and their Children’s Health
4d-WFR	4 day weighed food records
WHO	World Health Organization
