# Supplementary material for: Methyl-Donor and Cofactor Nutrient Intakes in the First 2–3 Years and Global DNA Methylation at Age 4: A Prospective Cohort Study
Source: Nutrients. 2018 Feb 27;10(3):273. doi: 10.3390/nu10030273 (PMC5872691; doi:10.3390/nu10030273)
Supplement: Supplementary file 1 [file nutrients-10-00273-s001.pdf]

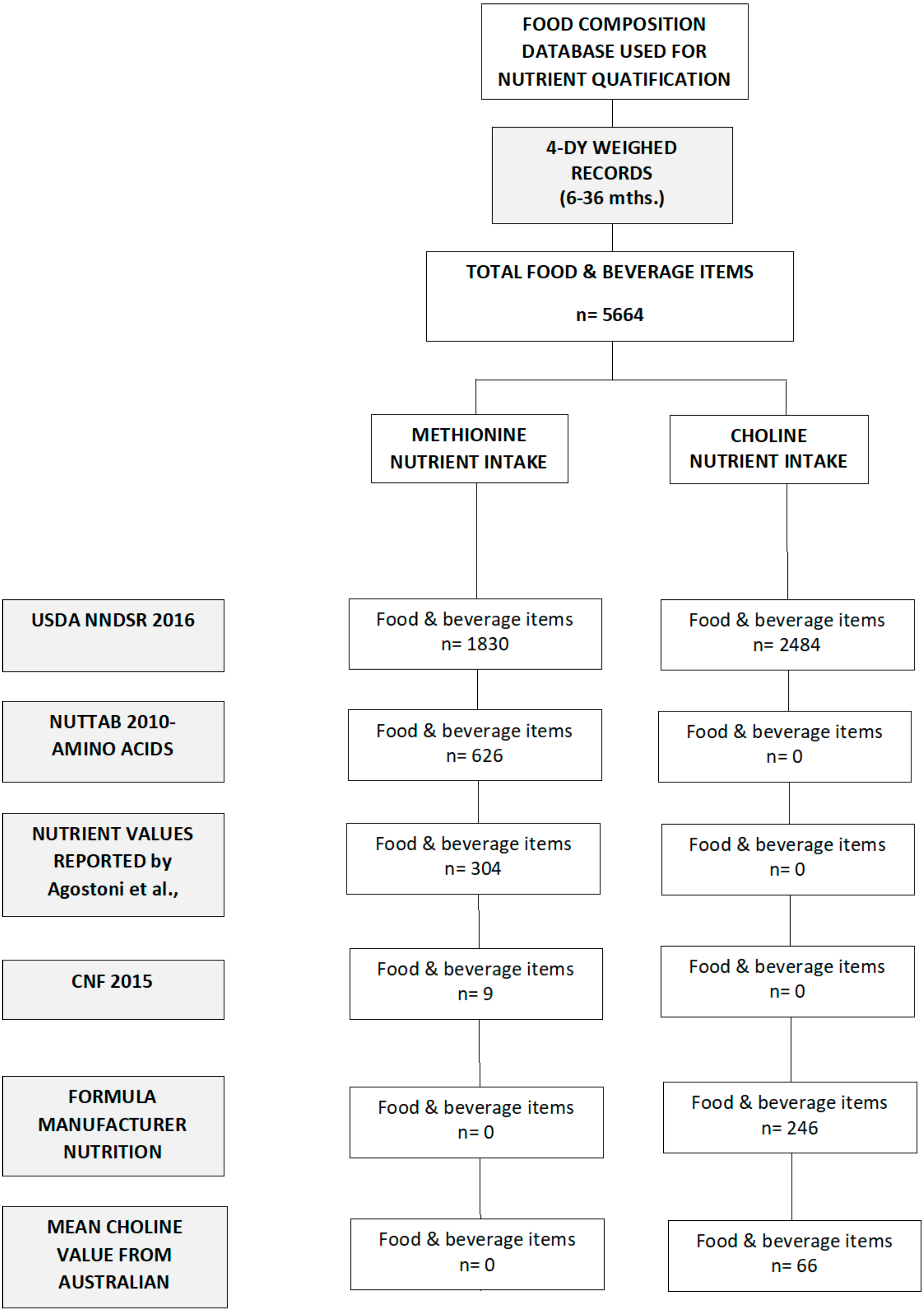


**Figure S1.** Food composition database used for methionine and choline quantification: 4-day weighed food records

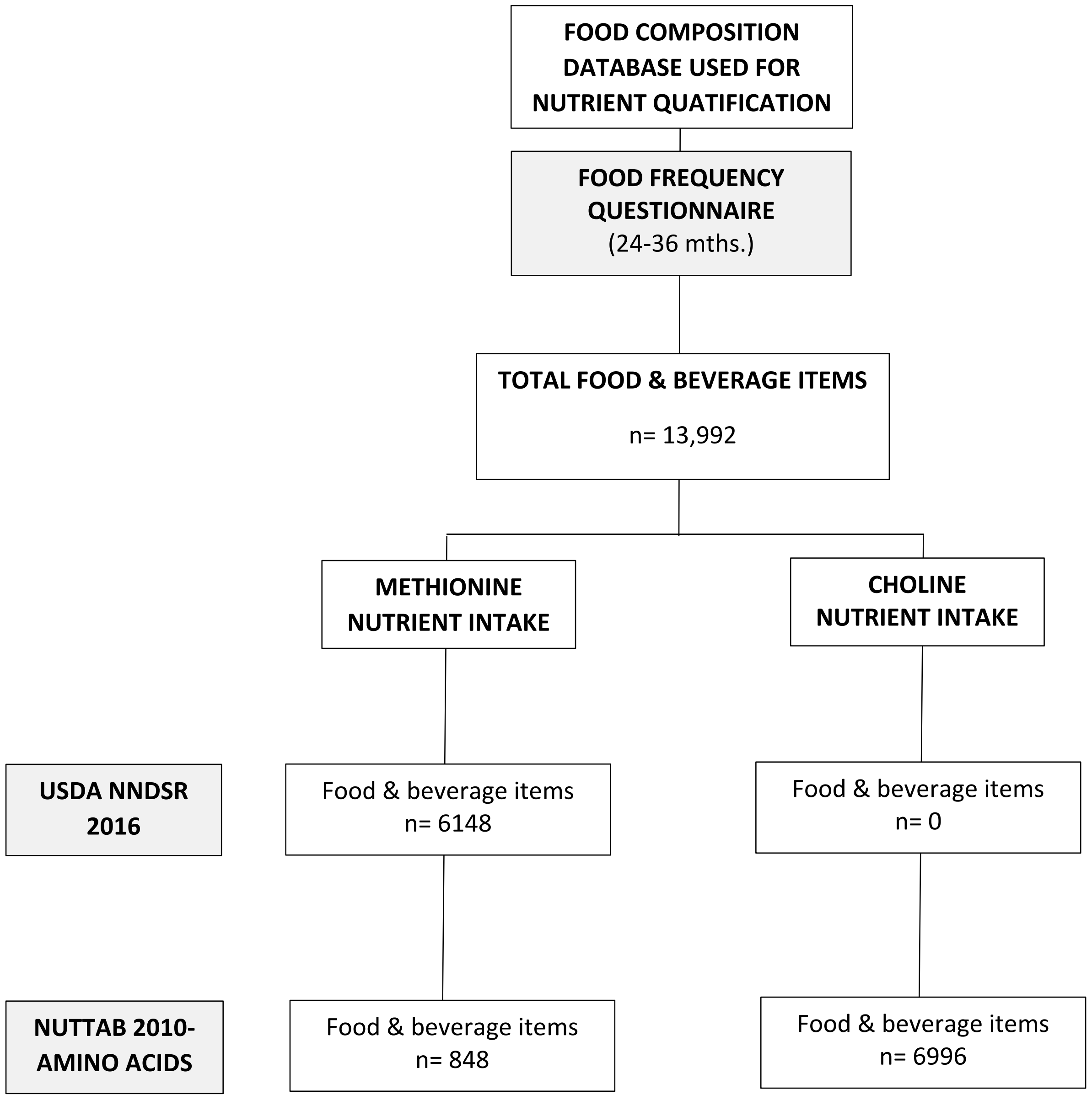


**Figure S2.** Food composition database used for methionine and choline quantification: food frequency questionnaires

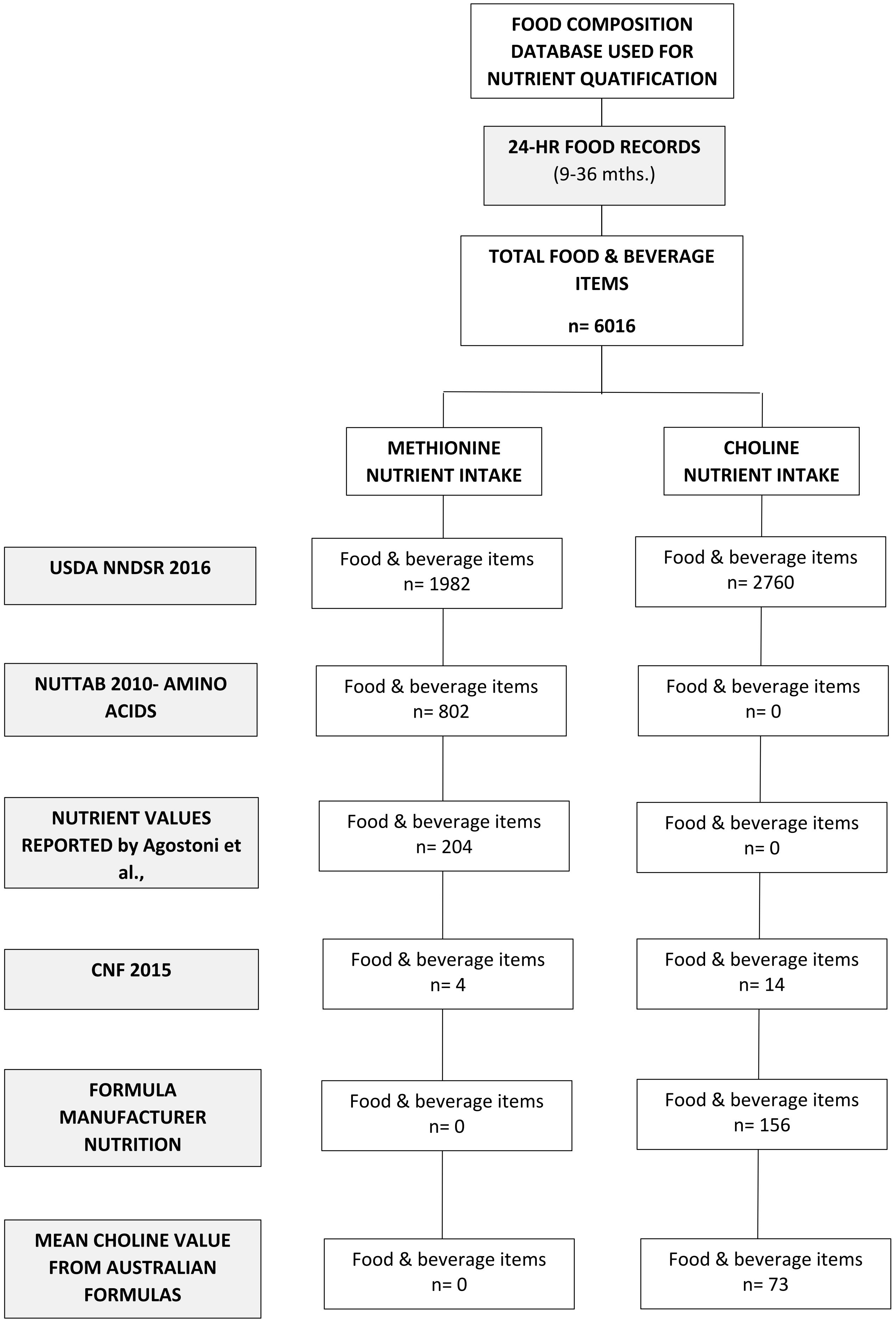


**Figure S3.** Food composition database used for methionine and choline quantification: 24-hour food recall

**Table S1. Quintiles of nutrient intake during early childhood in the WATCH cohort**

| | | Quintile range (minimum, maximum) at each time point (months) | | | | | |
|---|---|---|---|---|---|---|---|
| **Nutrient (Total daily intake)** | **QR** | **3** | **6** | **9** | **12** | **24** | **36** |
| Methionine, mg (Amino acid) | 1 | (1.6, 2.3) | (5.0, 123.0) | (3.5, 202.6) | (11.1, 384.9) | (470.7, 704.3) | (444.1, 743.1) |
| | 2 | (2.6, 156.8) | (128.0, 167.1) | (235.5, 355.0) | (387.5, 495.2) | (756.4, 1001.6) | (811.7, 974.9) |
| | 3 | (156.8, 173.3) | (174.0, 220.6) | (381.3, 458.0) | (558.9, 874.5) | (1060.7, 1165.3) | (1000.7, 1263.0) |
| | 4 | (176.4, 193.3) | (231.2, 267.8) | (462.2, 540.5) | (891.3, 1120.9) | (1219.2, 1404.7) | (1278.3, 1478.2) |
| | 5 | (195.1, 318.3) | (290.5, 726.1) | (545.6, 1259.0) | (1204.1, 2651.9) | (1404.9, 1696.2) | (1503.5, 2262.8) |
| Vit. B2, mg (Riboflavin) | 1 | (0.1, 0.2) | (0.1, 0.3) | (0.1, 0.4) | (0.2, 1.0) | (0.9, 1.4) | (0.3, 1.3) |
| | 2 | (0.2, 0.2) | (0.3, 0.3) | (0.5, 0.6) | (1.0, 1.7) | (1.4, 1.7) | (1.3, 1.7) |
| | 3 | (0.2, 0.3) | (0.3, 0.4) | (0.7, 1.1) | (1.8, 2.7) | (1.8, 2.3) | (1.8, 2.2) |
| | 4 | (0.3, 0.3) | (0.4, 0.7) | (1.2, 1.5) | (2.9, 4.1) | (2.3, 3.2) | (2.2, 3.0) |
| | 5 | (0.3, 1.4) | (0.7, 1.4) | (1.5, 4.3) | (4.1, 9.5) | (3.4, 610.4) | (3.1, 4.6) |
| Vit. B6, mg (Pyridoxine) | 1 | (0.0, 0.1) | (0.1, 0.1) | (0.1, 0.3) | (0.1, 0.5) | (0.0, 0.1) | (0.0, 0.3) |
| | 2 | (0.1, 0.1) | (0.1, 0.3) | (0.3, 0.4) | (0.5, 0.6) | (0.2, 0.4) | (0.3, 0.6) |
| | 3 | (0.1, 0.1) | (0.3, 0.4) | (0.4, 0.5) | (0.7, 0.8) | (0.4, 0.6) | (0.6, 0.7) |
| | 4 | (0.1, 0.4) | (0.4, 0.5) | (0.5, 0.6) | (0.8, 1.0) | (0.6, 0.8) | (0.7, 1.0) |
| | 5 | (0.4, 0.6) | (0.5, 0.7) | (0.6, 1.7) | (1.0, 2.1) | (0.9, 1.8) | (1.2, 2.8) |
| Vit. B12, µg (Cobalamin) | 1 | * | (0.0, 0.0) | (0.0, 0.4) | (0.0, 1.3) | (0.6, 1.7) | (0.9, 2.2) |
| | 2 | (0.0, 0.0) | (0.0, 0.1) | (0.5, 1.1) | (1.3, 2.0) | (2.0, 3.2) | (2.2, 2.8) |
| | 3 | * | (0.1, 0.6) | (1.2, 1.7) | (2.0, 2.8) | (3.5, 4.9) | (2.8, 3.6) |
| | 4 | (0.1, 1.2) | (0.6, 1.6) | (1.7, 2.2) | (3.0, 4.9) | (5.0, 7.1) | (3.7, 5.6) |
| | 5 | (1.3, 2.0) | (1.6, 2.5) | (2.4, 4.5) | (5.1, 7.8) | (7.3, 11.6) | (5.7, 16.8) |
| Choline, mg | 1 | (12.1, 88.0) | (58.4, 95.3) | (84.2, 109.0) | (80.5, 116.4) | (108.4, 142.4) | (63.0, 127.5) |
| | 2 | (88.0, 99.7) | (95.6, 116.4) | (114.7, 126.1) | (120.2, 141.5) | (147.0, 173.2) | (130.7, 168.5) |
| | 3 | (99.8, 108.8) | (117.2, 130.0) | (129.4, 138.0) | (143.6, 162.8) | (174.5, 202.2) | (182.2, 204.9) |
| | 4 | (110.5, 120.3) | (131.5, 144.2) | (139.2, 157.6) | (163.5, 198.9) | (203.1, 240.6) | (205.6, 238.1) |
| | 5 | (123.5, 175.0) | (155.5, 415.7) | (157.8, 308.7) | (199.7, 339.1) | (240.9, 440.7) | (240.3, 385.0) |
| Folate, µg | 1 | (19.9, 31.2) | (18.6, 44.7) | (27.8, 66.1) | (35.3, 109.2) | (125.3, 179.3) | (80.9, 190.7) |
| | 2 | (31.3, 35.2) | (47.4, 51.6) | (69.1, 93.0) | (111.7, 196.6) | (201.5, 222.5) | (190.7, 239.4) |
| | 3 | (35.4, 38.7) | (52.1, 65.7) | (98.5, 120.6) | (206.6, 313.0) | (228.9, 270.6) | (248.1, 345.0) |
| | 4 | (38.8, 65.0) | (69.5, 92.5) | (121.4, 209.0) | (358.7, 597.1) | (292.1, 398.7) | (362.6, 477.3) |
| | 5 | (70.4, 101.4) | (96.9, 216.0) | (211.8, 722.3) | (617.7, 1602.3) | (434.2, 812.1) | (496.9, 1367.2) |

* Vitamin B12 had numerous zero values for the 3 month time period, therefore all grouped into rank value = 2
QR: quintile rank, Vit: vitamin
